# Androgens Tend to Be Higher, but What about Altered Progesterone Metabolites in Boys and Girls with Autism?

**DOI:** 10.3390/life12071004

**Published:** 2022-07-07

**Authors:** Benedikt Gasser, Johann Kurz, Genevieve Escher, Hiten D. Mistry, Markus G. Mohaupt

**Affiliations:** 1 Department of Movement, Science and Sports, University of Basel, 4052 Basel, Switzerland; 2 Department of Clinical Research, University of Bern, 3010 Berne, Switzerland; genevieve.escher@dbmr.unibe.ch; 3 Intersci Research Association, Karl Morre Gasse 10, 8430 Leibnitz, Austria; john@a1.net; 4 Department of Women and Children’s Health, School of Life Course and Population Health Sciences, King’s College London, London WC2R 2LS, UK; hiten.mistry@kcl.ac.uk; 5 Teaching Hospital Internal Medicine, Lindenhofgruppe, 3006 Berne, Switzerland; markus.mohaupt@lindenhofgruppe.ch

**Keywords:** progesterone metabolites, androgen theory of autism, gender ratio of autism

## Abstract

Background: Evidence exists that steroid hormones are altered in individuals with autism, especially androgens. Despite lower prevalence in girls than boys, evidence of potential alterations in progesterone metabolites is sparse, so the aim of this study was to elucidate different progesterone metabolites in affected children with autism versus healthy controls. Material and Methods: Circadian urine samples from 48 boys and 16 girls with autism spectrum disorders and a matched case–control group were analysed for progesterone metabolites by gas chromatography–mass spectrometry and normalised for creatinine excretion. Results: In boys with autism, the majority of progesterone metabolites were reduced, such as progesterone, 6a-OH-3a5b-TH-progesterone, or 20a-DH-progesterone (*p* < 0.01 for all). In girls with autism, a similar pattern of reduction in progesterone metabolites was detected; however, potentially due to the relatively small sample, this pattern was only detectable on the level of a trend. Discussion: As stated, androgen levels are higher in boys and girls with autism, but evidence for progesterone metabolites is much sparser. The pattern of a decrease in progesterone metabolites suggests the existence of an altered routing of steroid metabolites, probably in combination with a dysregulation of the HPAG axis. As, recently, increased CYP17A1 activity has been suggested, the stronger routing towards androgens is further implied in line with our findings of lower progesterone concentrations in boys and girls with autism than healthy controls.

## 1. Introduction

**Figure 1:**
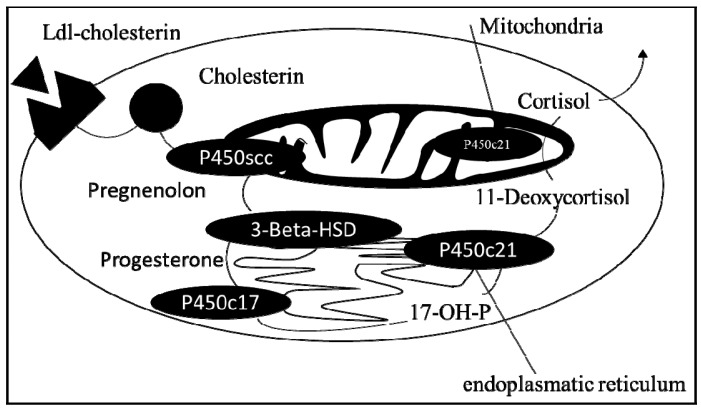
From acetate via citrate cyclus to cholesterol as precursor of progesterone. Interestingly, progesterone is not only metabolized in the adrenal gland and sex organs but also in the kidney and liver. Focusing on progesterone synthesis, conversion from cholesterol to pregnenolone takes place in mitochondria through a P450-enzyme complex (hydroxylase/desmolase) which is induced through ACTH, linking the metabolite synthesis pathway with the HPA axis, implying that progesterone acts as a prohormone for androgens and oestrogens [31,36,37].

Increasing evidence exists for a dysregulation of the hypothalamic–pituitary–adrenal axis (HPA axis) in autism and thus of adrenal gland mineralocorticoid, glucocorticoid, and androgen metabolites [1,2,3,4,5,6]. These metabolites are characterized through cholesterol as precursor, suggesting that all classes of steroid hormones are altered in autism. Thereby, a link between cholesterol, vitamin D, and steroid hormones subsequently impacting the development of autism has been suggested [7,8,9] (Figure 1). Hints of an involvement of progesterone and oestrogen metabolites in autism exist, but evidence is still sparse [1,9,10]. This is needed, as prevalence of autism in girls is much lower than boys. Progesterone has several effects on the central nervous system during human development. Cyclical secretion also released in response to stress, suggesting an important and dynamic role in social affiliation [11]. The clearly increased prevalence of autism in boys compared to girls has led to speculations about the association of sex hormones with autism; however, androgens especially have been the core of interest [1,10,12,13]. This is not wrong, as several lines of evidence point to androgens, such as an association of autism with fragile X-syndrome, extreme male theory of autism, or even one of the first descriptions of autism by Hans Asperger with an involvement of testes [1,14,15]. Generally, as a female phenotype is dependent on progesterone metabolites, a principal involvement for autism is implied [16]. Mothers of children with autism reported low progesterone levels in obstetric records, suggesting that low progesterone may be contributing to changes in the brain associated with autism [16]. Furthermore, progesterone and oestrogen used in oral contraceptives have been reported to modify the condition of oocytes and give rise to a potent risk factor that might explain the recent increase in the prevalence of autism [17,18]. Progesterone was indicated to influence hirsutism, bisexuality or asexuality, irregular menstrual cycle, dysmenorrhea, polycystic ovary syndrome, severe acne, and family history of ovarian, uterine, and prostate cancer, and interestingly, women with autism reported significantly more often such symptoms [13]. Furthermore, precocious puberty is associated with steroid hormone dysregulations and a higher occurrence of autism [19]. Furthermore, a role of mini-puberty is likely to have relevance for neurodevelopmental disorders, such as autism [20,21]. Although very recent evidence suggests that differences in neurosteroid levels play a role in generating different disease phenotypes in autism, evidence of the manner and levels of alterations of these hormones is still not finally elucidated [22,23,24,25]. Nevertheless, different lines of evidence highlight the practical relevance of progesterone metabolites for autism. Recently, progesterone metabolites were identified as part of the neuroendocrine basis of social bonds and enable individuals to suppress self-interests when necessary in order to promote the well-being of another person, whereas lower levels of progesterone tend to be inversely associated with behavioural traits found in autism [26,27]. More precisely, it was also shown that bonding with others increases levels of progesterone as a further index of involvement of progesterone metabolites in autism [27]. In addition, psychosomatic research demonstrated that persisting stress in females led to delayed puberty, decreased levels of progesterone in exchange for cortisol, increased incidence of ovulatory cycles, impaired implantation, greater risk of miscarriage, prolonged interbirth intervals, and accelerated reproductive senescence [28]. Furthermore, for progesterone, it was indicated to influence neuronal migration in subcortical, periventricular, hippocampal, and cerebellar heterotopias of autistic subjects, leading to abnormal neuronal migration [29,30,31]. Motor impairments are further signs of autism, allowing a link for these findings with typical clinical pathology [29]. Interactions were suggested through regulation in the form of an angiogenesis cascade where progesterone inhibits ACTH, which normally upregulates VEGF-A in humans [30,31,32,33,34,35]. Effects of angiogenesis would frame the development of an autistic phenotype in line with the understanding of autism as a pervasive development disorder.

To summarize, androgen and progesterone exposure is highly relevant for gender differentiation [38]. As around a four times higher prevalence of autism in boys than girls, a role is implied. However, all these presented facts of progesterone metabolites with autism evidence of the alterations in these in affected subjects with autism is still sparse, which led directly to the aim of this study of clearly characterizing progesterone metabolites in a clinical cohort of boys and girls with autism as compared to healthy controls. As a hypothesis with potential falsification, it shall be stated that no differences can be detected in progesterone metabolites between children with autism as compared to normally developed children [39].

## 2. Material and Methods

### 2.1. Study Design

This study was carried out in accordance with the Declaration of Helsinki. After the study procedures had been fully explained, the parents of participants read and signed informed consent forms. Individuals were included after they had given written informed consent, which was also signed by their legal guardians. In the control group, autism was excluded by the Marburg Questionnaire for Asperger Syndrome (MBAS), fulfilled by the caregivers. The study was approved by the Government Ethics Board of Graz, Austria (Approval Number FA8B-50.2) and registered at ClinicalTrials.gov ID NCT01197131. Autistic and control white European boys and girls were recruited from the area of Graz (Austria), mid-2009 to mid-2012. Participants were excluded if they had a neurological and psychiatric disorder other than autism and comorbid disorders; history of liver diseases, renal or endocrine disorders, current infection, or fever. Mental retardation or behavioural disorders were exclusion criteria only for the control group, but were allowed as comorbid conditions in the autistic group.

### 2.2. Clinical Evaluation

Each autistic child was diagnosed by experienced medical doctors accustomed to clinical symptoms of autism according to diagnostic criteria of ICD-10 (International Classification of Diseases, 10th Revision) from the World Health Organisation (WHO) and DSM-IV (*Diagnostic and Statistical Manual of Mental Disorders*) and the MBAS.

### 2.3. Participants

Our sample consisted of 48 autistic boys and 16 autistic girls, as well as 60 healthy boys and 36 healthy girls (details of the cohort are shown in Supplemental Table S1).

### 2.4. Analysis of Urinary Steroids by Gas Chromatography–Mass Spectrometry

Urine samples were taken in the morning between 7:00 and 9:00 am after breakfast. Urine sample preparation comprised pre-extraction, enzymatic hydrolysis, extraction from the hydrolysis mixture, derivatization, and gel filtration, as several times described by us and others [40,41]. The recovery standard was prepared by adding 2.5 μg of medroxyprogesterone to 1.5 mL of urine. The sample was extracted on a Sep-Pak C18 column (Waters Corp., Milford, MA, USA), dried, reconstituted in 0.1 M acetate buffer, pH 4.6, and hydrolysed with powdered Helix pomatia enzyme (12.5 mg; Sigma Chemical Co., St. Louis, MO, USA) and 12.5 μL of β-glucuronidase/arylsulfatase liquid enzyme (Roche Diagnostics, Rotkreuz, Switzerland). The resulting free steroids were extracted on a Sep-Pak C18 cartridge. A mixture of internal standards (2.5 μg each of 5α-androstane-3α, 17α-diol, stigmasterol, and cholesterol butyrate, and 0.15 μg of 3β5β-tetrahydroaldosterone) was added to this extract and the sample derivatized to form the methyloxime-trimethylsilyl ethers. Analyses were performed on a Hewlett Packard gas chromatograph 6890 (Hewlett Packard, Palo Alto, CA, USA) with a mass selective detector 5973 by selective ion monitoring (SIM). One characteristic ion was chosen for each compound measured. The derivatized samples were analysed during a temperature-programmed run (210–265 °C) over a 35-min period. The calibration standard consisted of a steroid mixture containing known quantities of all steroid metabolites to be measured. Responses and retention times were recorded regularly. In each case, the ion peak was quantified against the internal stigmasterol standard. With this method, the most relevant metabolites of the glucocorticoid synthesis pathway were measured and presented. All results were adjusted for creatinine in urine to check renal function.

### 2.5. Statistical Analysis

Group comparisons were performed for the subsample of 48 boys with autism versus first all 60 healthy boys and second an individually pairwise matched cohort for age and BMI of 48 boys (pairing). For the first group, differences were analysed with two-sided heteroscedastic *t*-tests. For the individually pairwise matched design, two-sided paired *t*-tests were performed. The same procedure was performed for the first 16 girls with autism versus 36 healthy girls and second for an individually pairwise matched sample of 16 healthy girls. Bonferroni correction for multiple comparison was applied. Furthermore, in order to detect an effect of age and BMI, a multivariate regression for the subsamples of all 48 boys and 16 girls with autism, as well as 60 healthy boys and 36 healthy girls for all progesterone metabolites was performed in the form: concentration of progesterone metabolite concentration_:_ = α ∗ BMI_i_ + β ∗ age_i_ + ε. Results with *p* < 0.05 were considered significant (*) and highly significant with *p* < 0.01 (**). Data are presented as mean ± SEM (standard error of the mean). Analyses were conducted with GraphPad Prism (GraphPad Software, Inc., La Jolla, CA, USA), Microsoft Excel (Microsoft Inc., Redmond, WA, USA), and SPSS (IBM Inc., Armonk, NY, USA).

## 3. Results

Three times as many boys as girls were included in the study, thus representing a typical gender distribution of autism spectrum diseases [1]. Analyses were conducted for all boys with autism (*n* = 48) versus all boys (*n* = 60) measured. Furthermore, paired matching was performed for boys, whereby no significant difference could be detected for age (*p* = 0.99) and BMI (*p* = 0.98) in boys. The same pattern applied in girls for age (*p* = 0.72) and BMI (*p* = 0.27). Furthermore, multivariate regressions of age and BMI on each progesterone concentration in the respective subsample (boys with autism, girls with autism, healthy boys, healthy girls) indicated no direct association. For age and BMI, in most cases, no significant estimators (Supplemental Table S3) could be identified. For example, in boys with autism only for 3a5a-TH-progesterone, 20a-DH-5a-DH-progesterone, and 20b-DH-progesterone significant estimators can be identified. The same pattern applies for girls with autism, whereby only a significant estimator was detected for 3a5a-TH-progesterone. In healthy boys, 20a-DH-3b5a-TH-progesterone was the only significant association for age. In healthy girls, this was for 3a5a-TH-progesterone for the intercept and age and for progesterone for the intercept.

The urinary concentrations (creatinine adjusted) are shown for the different progesterone metabolites separated for boys and girls versus all healthy controls in Table 1 and for the individually pairwise matched samples in Supplemental Table S2. In boys with autism, the majority of progesterone metabolites were altered (e.g., progesterone, 6a-OH-3a5b-TH-progesterone, 20a-DH-progesterone (*p* < 0.01 for all) and the average of all progesterone metabolites (*p* < 0.01)) for the analyses of all subjects included in the study. In boys, only 17a20a-DH-progesterone, 6a-OH-progesterone, and 20a-DH-5a-DH-progesterone were not altered on an alpha = 0.1 level. All other progesterone metabolites were lower. A similar pattern of alterations of progesterone metabolites was found when using the statistical design with individually pairwise matching for age and BMI (attempt of twin matching) (Supplemental Table S1). In girls with autism, progesterone was reduced; however, the other metabolites did not reach statistical significance at an alpha level smaller than 0.01, despite the sum of all metabolites (*p* < 0.01). As in boys, a similar pattern of alterations was achieved with an individually pairwise matching (Supplemental Table S1).

## 4. Discussion

Our results showed alterations in progesterone metabolites in children with autism as compared to the matched healthy controls, allowing us to falsify the initially stated hypothesis of no alterations in progesterone metabolites in subjects with autism versus healthy controls [39]. As we were glad to find children with autism to enrol in the study, these were mainly from families interested in the subject motivated to support our effort to resolve the enigma of autism. In consequence, we chose an approach as simple as possible. One main limitation of the study was that we did not report Tanner stages. Undoubtedly, the best way for matching would have been to use Tanner stages, as these most accurately indicate puberty status. Here, we only show the results of group comparisons of a sample of boys and girls with autism versus healthy controls. Furthermore, we attempted to solve the problem of a potential bias due to age and BMI with an individually pairwise matched design (attempt of twins matching), whereby from the pool of healthy children, the most similar healthy child for age and BMI was compared to a child with autism. Despite this approach, the pattern of alterations remained almost the same. However, as age and BMI are only indicative for pubertal status, a potential bias might still exist. Nevertheless, when summarizing the findings from the multivariate regression models almost no significant relationship between age and BMI and progesterone concentrations of a respective metabolite could be detected. This supports the assumption, that one main effect on the respective progesterone concentrations is the diagnosis of autism. This suggests that these alterations might be beside other factors, e.g., increased substrate availability in line with the cholesterol theory of autism [7] due to a general dysregulation of the HPAG axis [6]. It suggests that the reduced progesterone metabolites led to less inhibition of ACTH and an effect of adrenal gland metabolites via VEGF on angiogenesis and secondary development of the central nervous system [33,34,35]. That the CRH-ACTH system was altered in affected subjects with autism was several times implied (reviewed by Taylor and Corbett) [6]. Furthermore, on a mechanistic level, such conditions have been described as enhancing coenzyme or electron transfer availability via stimulated oxidoreductase activity potentially caused by oxidative stress to excess androgen exposure, despite otherwise normal regulatory feedback responses [42,43,44,45,46]. What remains unclear is the fact that results are once again less concise for girls than boys. Here, as often in the analyses the smaller sample in girls potentially led to a lower power of the results in girls. However, we suggest, besides the sample size, another factor on receptor level plays a crucial role. It was shown that retinoic acid-related orphan receptor-alpha (RORA) regulates the enzyme aromatase (converting testosterone to oestrogen) [22,23,24,25]. As this would clearly explain higher androgen levels in combination with lower progesterone levels in affected children with autism, the gender bias and the alterations found here could be explained [22,23,24,25]. It was shown that aromatase activity was significantly reduced in the frontal cortex of individuals with autism [22,23,24,25]. A model for reciprocal hormonal effects on RORA of estrogens and androgens was postulated, in line with an observed reduction of RORA in autistic brains, which may lead to increased testosterone levels through downregulation of aromatase, whereby through the androgen receptor, testosterone negatively modulates RORA, whereas oestrogen upregulates RORA through the oestrogen receptor [22,23,24,25]. It was suggested that the expression of RORA is inversely modulated by male and female sex hormones, confirming our findings [22,23,24,25]. However, the specific mechanism and circumstances through which androgen and oestrogen receptors regulate RORA in opposite directions are unknown, and its interaction with progesterone metabolites require further study [22,23,24,25].

Focusing on the specific pattern of alteration in the metabolites measured, different effects of 5α- and 5β-reduction on the central nervous system are described, implying a complex network of steroid alterations influencing brain development [47]. In our measured sample 5α and 5β metabolites showed almost the same pattern of alteration of reduction in boys, making it hard to come to suggestions. Nevertheless, it is important to keep in mind that different epimers induce different biological actions, e.g., 5α-pregnan-3α-ol-20-one (3α-5α-THP) mainly acts to enhance postsynaptic GABA-ergic activity, while androgens enhance GABA-activated currents [48,49]. Different actions of steroids have been described for 5α-progestins depending mainly on gene action, while 5β-progestins act directly on excitable membranes by modifying membrane permeability [47,50]. Focusing on the synapse level, progesterone inhibits neuronal nicotinic acetylcholine receptors (nAChRs), whereas its 3α,5α-reduced metabolite 3α,5α-tetrahydroprogesterone (allopregnanolone) activates the type A gamma-aminobutyric acid (GABA_A_) receptor complex, indicating the different role of epimers [51]. Nevertheless, in subjects with autism, epimer differences could be responsible for the different autisms, in line with our understanding of autism as a spectrum [52].

To focus back on the pattern of a reduction in the most progesterone metabolites, we suggest that general overstimulation of different cholesterol-dependent substances, respectively, steroid hormone precursors with a strong routing towards androgens [7]. This would be in line with other reports, e.g., on vitamin D homeostasis for which cholesterol also act as a precursor [8,53]. Evidence was presented that vitamin D hormone activates the transcription of the serotonin-synthesizing gene tryptophan hydroxylase 2 in the brain at a vitamin D response element and represses the transcription of tryptophan hydroxylase 1 in tissue outside the blood–brain barrier at a distinct vitamin D response element [8,53]. Furthermore, the work by Gevi et al. [54] found that the tryptophan and purine metabolism was altered in affected subjects with autism, which has excess melatonin an influence on mitochondrial activity, as melatonin is suggested to reduce oxidative stress [55]. Measurements with highest differences performed belonged to the tryptophan and purine metabolic pathways. Also, vitamin B6, riboflavin, phenylalanine–tyrosine–tryptophan biosynthesis, pantothenate and CoA, and pyrimidine metabolism differed significantly [54]. It seems that affected subjects with autism transform tryptophan into xanthurenic acid and quinolinic acid (two catabolites of the kynurenine pathway), at the expense of kynurenic acid and especially of melatonin [54]. Gevi et al. therefore directly implied a large reduction in melatonin concentrations in affected subjects with autism and in consequence higher levels of oxidative stress, which might influence 17/20 lyase activity yielding lower levels of progesterone metabolites [43,54].

## Figures and Tables

**Table 1:** Progesterone metabolites for *n* = 48 boys with autism and *n* = 16 girls with autism versus a sample of *n* = 36 healthy girls.

Ratio of Metabolites (mmol/L) to	Urine Creatinine (μmol/L)	Girls with Autism	*p*-Value	Healthy Girls	Boys with Autism	*p*-Value	Healthy Boys
Chemical Name	Trivial Name	Mean ± SEM (μg/L)		Mean ± SEM (μg/L)	Mean ± SEM (μg/L)		Mean (μg/L) ± SEM
5a-Pregnan-3a-ol-20-one	3a5a-TH-progesterone	3.97 ± 0.15	0.215	11.42 ± 0.6	2.296 ± 0.256	0.017	3.245 ± 0.376
5a-Pregnan-3b-ol-20-one	3b5a-TH-progesterone	0.25 ± 0.02	0.472	0.21 ± 0.07	0.149 ± 0.035	0.054	0.148 ± 0.019
4-Pregnen-3,20-dione	Progesterone	0.99 ± 0.03	0.005	3.3 ± 0.13	1.014 ± 0.102	<0.001	2.796 ± 0.317
5a-Pregnan-3b,20a-diol	20a-DH-3b5a-TH-progesterone	1.78 ± 0.07	0.034	3.89 ± 0.26	1.545 ± 0.204	0.038	1.904 ± 0.155
5b-Pregnan-3a,6a-diol-20-one	6a-OH-3a5b-TH-progesterone	7.8 ± 0.42	0.134	16.93 ± 1.66	4.519 ± 0.387	0.004	7.196 ± 0.898
5a-Pregnan-20a-ol-3-one	20a-DH-5a-DH-progesterone	4.46 ± 0.36	0.379	3.33 ± 1.42	3.72 ± 0.798	0.332	4.011 ± 1.101
4-Pregnen-20b-ol-3-one	20b-DH-progesterone	0.97 ± 0.06	0.834	1.42 ± 0.22	0.436 ± 0.057	0.016	0.607 ± 0.061
4-Pregnen-20a-ol-3-one	20a-DH-progesterone	1.35 ± 0.04	0.327	1.51 ± 0.17	2.992 ± 0.61	<0.001	0.905 ± 0.126
4-Pregnen-6b-ol-3,20-dione	6b-OH-progesterone	1.65 ± 0.12	0.352	1.79 ± 0.46	0.998 ± 0.118	0.048	1.451 ± 0.237
4-Pregnen-11a-ol-3,20-dione	11a-OH-progesterone	14.55 ± 0.53	0.327	11.88 ± 2.12	8.847 ± 0.786	0.084	10.325 ± 0.968
4-Pregnen-6a-ol-3,20-dione	6a-OH-progesterone	5.24 ± 0.42	0.234	5.46 ± 1.69	3.833 ± 0.711	0.342	4.366 ± 0.859
4-Pregnen-17,20a-diol-3-one	17a20a-DH-progesterone	5.6 ± 0.27	0.407	9.32 ± 1.09	5.488 ± 0.785	0.631	5.42 ± 0.789
Average of Sum of al progesterone metabolits for an individual		48.61 ± 2.49	*p* < 0.01	67.13 ± 9.89	35.837 ± 4.89	*p* < 0.01	42.374 ± 5.906

## Supplementary Materials

The following supporting information can be downloaded at: https://www.mdpi.com/article/10.3390/life12071004/s1, Table S1: Characteristics of the clinical cohort; Table S2: Analysis of the individually pairwise matched cohort of 48 boys with autism and 16 girls with autism; Table S3: Results of the multivariate regression for the subsamples of boys and girls with autism as well as controls for all progesterone metabolites yielding: concentration of progesterone metabolite concentrationi = α ∗ BMIi + β ∗ agei + ε. Significant estimators are marked in green.
