# Supplementary material for: Androgens Tend to Be Higher, but What about Altered Progesterone Metabolites in Boys and Girls with Autism?
Source: Life (Basel). 2022 Jul 7;12(7):1004. doi: 10.3390/life12071004 (PMC9324026; doi:10.3390/life12071004)
Supplement: Supplementary file 1 [file life-12-01004-s001.zip › life-1707296-supplementary.pdf]

Supplemental Table S1. Characteristics of the clinical cohort.

Boys	BMI [kg/m^2]	Age [y]
autism (n = 48)	19.1 ± 0.6	14.2 ± 0.5
all healthy (n = 60)	17.6 ± 3.6	12.9 ± 5.9
individually pairwise matched (n = 48)	18.6 ± 0.3	14.3 ± 0.5
Girls		
autism (n = 16)	17.5 ± 0.7	13.8 ± 1.0
all healthy (n = 36)	17.5 ± 2.7	13.6 ± 3.2
individually pairwise matched (n = 16)	17.2 ± 0.8	13.2 ± 0.8

Supplemental Table S2. Analysis of the individually pairwise matched cohort of 48 boys with autism and 16 girls with autism.

Ratio of metabolites (mmol/l) to<br>Chemical Name	Urine creatinine (micromol/l)<br>Trivial Name	Girls with autism<br>Mean ± SEM (µg/l)	p-Value	Healthy girls<br>Mean ± SEM (µg/l)	Boys with autism<br>Mean ± SEM (µg/l)	p-Value	Healthy Boys<br>Mean (µg/l) ± SEM
5a-Pregnan-3a-ol-20-one	3a5a-TH-progesterone	3.97 ± 0.15	0.74	4.17 ± 0.39	2.296 ± 0.256	<0.001	6.92 ± 0.26
5a-Pregnan-3b-ol-20-one	3b5a-TH-progesterone	0.25 ± 0.02	0.3	0.18 ± 0.027	0.149 ± 0.035	0.48	0.18 ± 0.027
4-Pregnen-3,20-dione	Progesterone	0.99 ± 0.03	0.016	2.69 ± 0.65	1.014 ± 0.102	<0.001	2.14 ± 0.18
5a-Pregnan-3b,20a-diol	20a-DH-3b5a-TH-progesterone	1.78 ± 0.07	0.045	3.65 ± 0.79	1.545 ± 0.204	0.397	1.35 ± 0.10
5b-Pregnan-3a,6a-diol-20-one	6a-OH-3a5b-TH-progesterone	7.8 ± 0.42	0.174	14.85 ± 4.82	4.519 ± 0.387	0.99	4.52 ± 0.54
5a-Pregnan-20a-ol-3-one	20a-DH-5a-DH-progesterone	4.46 ± 0.36	0.4	3.33 ± 0.82	3.72 ± 0.798	<0.001	8.81 ± 1.89
4-Pregnen-20b-ol-3-one	20b-DH-progesterone	0.97 ± 0.06	0.74	1.05 ± 0.15	0.436 ± 0.057	0.54	0.39 ± 0.05
4-Pregnen-20a-ol-3-one	20a-DH-progesterone	1.35 ± 0.04	0.34	1.1 ± 0.18	2.992 ± 0.61	<0.001	1.05 ± 0.15
4-Pregnen-6b-ol-3,20-dione	6b-OH-progesterone	1.65 ± 0.12	0.51	1.56 ± 0.5	0.998 ± 0.118	0.45	2.77 ± 0.65
4-Pregnen-11a-ol-3,20-dione	11a-OH-progesterone	14.55 ± 0.53	0.73	16.15 ± 3.39	8.847 ± 0.786	<0.001	10.82 ± 0.68
4-Pregnen-6a-ol-3,20-dione	6a-OH-progesterone	5.24 ± 0.42	0.95	5.16 ± 0.89	3.833 ± 0.711	<0.001	4.54 ± 1.03
4-Pregnen-17,20a-diol-3-one	17a20a-DH-progesterone	5.6 ± 0.27	0.33	6.97 ± 1.2	5.488 ± 0.785	0.12	4.90 ± 0.64
Sum of al progesterone metabolites		48.61 ± 2.49	0.32	59.57 ± 10.13	35.837 ± 4.89	<0.001	41.72 ± 5.60

Supplemental Table S3. Results of the multivariate regression for the subsamples of boys and girls with autism as well as controls for all progesterone metabolites yielding: concentration of progesterone metabolite $concentration_i = \alpha * BMI_i + \beta * age_i + \varepsilon$. Significant estimators are marked in green.

Autistic boys	Coefficient of determination (R^2)	Intercept	p-value	BMI	p-value	Age	p-value
3a5a-TH-progesterone	0.273	-54.156	0.028	0.005	0.997	6.128	0.001
3b5a-TH-progesterone	0.084	-0.268	0.861	-0.054	0.519	0.224	0.052
Progesterone	0.016	7.241	0.351	-0.052	0.902	0.448	0.434
20a-DH-3b5a-TH-progesterone	0.057	0.415	0.975	-0.113	0.874	1.413	0.146
6a-OH-3a5b-TH-progesterone	0.008	5.292	0.008	0.018	0.862	-0.080	0.576
20a-DH-5a-DH-progesterone	0.176	-109.604	0.170	-4.001	0.359	17.588	0.004
20b-DH-progesterone	0.135	-7.195	0.217	0.004	0.991	0.951	0.029
20a-DH-progesterone	0.052	-19.841	0.575	1.545	0.427	1.829	0.483
11a-OH-progesterone	0.035	12.111	0.003	0.088	*0.674*	-0.354	0.221
6a-OH-progesterone	0.156	-100.746	0.055	4.421	0.123	4.529	0.236
17a20a-DH-progesterone	0.047	-3.375	0.941	0.939	0.710	3.419	0.315
Sum	0.112	11.410	0.303	0.278	0.641	1.359	0.102

Autistic girls	Coefficient of determination (R^2)	Intercept	p-value	BMI	p-value	Age	p-value
3a5a-TH-progesterone	0.686	-6.725	0.017	0.489	0.049	0.178	0.262
3b5a-TH-progesterone	0.354	-0.029	0.941	-0.025	0.490	0.050	0.055
Progesterone	0.028	1.289	0.189	-0.047	0.591	0.035	0.555
20a-DH-3b5a-TH-progesterone	0.249	5.195	0.009	-0.241	0.143	0.044	0.678
6a-OH-3a5b-TH-progesterone	0.082	-4.608	0.701	0.905	0.414	-0.198	0.788
20a-DH-5a-DH-progesterone	0.111	-0.856	0.932	-0.126	0.892	0.530	0.400
20b-DH-progesterone	0.745	-3.022	0.003	0.163	0.052	0.090	0.104
20a-DH-progesterone	0.406	-1.176	0.241	0.138	0.139	0.015	0.802
11a-OH-progesterone	0.135	9.939	0.505	1.322	0.337	-1.255	0.183
6a-OH-progesterone	0.152	7.069	0.548	-0.973	0.369	1.036	0.165
17a20a-DH-progesterone	0.011	2.656	0.745	0.205	0.784	-0.036	0.943
Sum	0.135	9.731	0.760	1.811	0.536	0.488	0.803

Control boys	Coefficient of determination (R^2)	Intercept	p-value	BMI	p-value	Age	p-value
3a5a-TH-progesterone	0.020	3.556	0.033	-0.057	0.330	0.015	0.876
3b5a-TH-progesterone	0.059	0.270	0.005	-0.004	0.211	-0.004	0.495
Progesterone	0.017	4.010	0.012	-0.034	0.542	-0.040	0.667
20a-DH-3b5a-TH-progesterone	0.110	3.563	0.000	0.017	0.516	-0.105	0.017
6a-OH-3a5b-TH-progesterone	0.028	12.426	0.006	0.029	0.854	-0.302	0.244
20a-DH-5a-DH-progesterone	0.008	0.973	0.858	-0.022	0.912	0.199	0.541
20b-DH-progesterone	0.005	0.520	0.077	-0.005	0.662	0.007	0.671
20a-DH-progesterone	0.024	1.228	0.052	-0.022	0.317	-0.002	0.959
11a-OH-progesterone	0.006	10.512	0.029	-0.088	0.603	0.068	0.809
6a-OH-progesterone	0.013	4.887	0.252	-0.123	0.421	0.069	0.785
17a20a-DH-progesterone	0.057	*2.452*	*0.476*	-0.182	0.144	0.284	0.169
Sum	0.021	44.398	0.002	-0.491	0.311	0.189	0.813

Control girls	Coefficient of determination (R^2)	Intercept	p-value	BMI	p-value	Age	p-value
3a5a-TH-progesterone	0.253	-78.594	0.042	0.915	0.735	5.428	0.023
3b5a-TH-progesterone	0.195	-0.157	0.374	0.004	0.733	0.021	0.056
Progesterone	0.060	8.171	0.024	-0.223	0.378	-0.071	0.738
20a-DH-3b5a-TH-progesterone	0.086	1.407	0.751	-0.214	0.505	0.456	0.100
6a-OH-3a5b-TH-progesterone	0.039	1.531	0.935	0.142	0.916	0.947	0.410
20a-DH-5a-DH-progesterone	0.086	1.407	0.751	-0.214	0.505	0.456	0.100
20b-DH-progesterone	0.186	-2.005	0.181	0.090	0.403	0.136	0.140
20a-DH-progesterone	0.213	-2.490	0.121	0.107	0.351	0.156	0.113

				-			
11a-OH-progesterone	0.038	25.247	0.051	0.535	0.556	-0.296	0.701
6a-OH-progesterone	0.327	-13.492	0.025	0.432	0.306	0.837	0.023
17a20a-DH-progesterone	0.027	-1.332	0.921	0.916	0.349	-0.391	0.635
Sum	0.139	-59.226	0.433	1.584	0.771	7.350	0.118
